# Future Directions: Analyzing Health Disparities Related to Maternal Hypertensive Disorders

**DOI:** 10.1155/2020/7864816

**Published:** 2020-08-01

**Authors:** Margaret Harris, Colette Henke, Mary Hearst, Katherine Campbell

**Affiliations:** ^1^Public Health Department, Henrietta Schmoll School of Health, St. Catherine University, St. Paul, MN 55105, USA; ^2^Department of Nursing, Henrietta Schmoll School of Health, St. Catherine University, St. Paul, MN 55105, USA; ^3^Department of Business Administration, School of Business, St. Catherine University, St. Paul, MN 55105, USA; ^4^Department of Interprofessional Education, Henrietta Schmoll School of Health, St. Catherine University, St. Paul, MN 55105, USA; ^5^Women's Health Integrative Research Center, Henrietta Schmoll School of Health, St. Catherine University, St. Paul, MN 55105, USA

## Abstract

Hypertensive disorders of pregnancy complicate up to 10% of pregnancies worldwide, constituting one of the most significant causes of maternal morbidity and mortality. Hypertensive disorders, specifically gestational hypertension, chronic hypertension, and preeclampsia, throughout pregnancy are contributors to the top causes of maternal mortality in the United States. Diagnosis of hypertensive disorders throughout pregnancy is challenging, with many disorders often remaining unrecognized or poorly managed during and after pregnancy. Moreover, the research has identified a strong link between the prevalence of maternal hypertensive disorders and racial and ethnic disparities. Factors that influence the prevalence of maternal hypertensive disorders among racially and ethnically diverse women include maternal age, level of education, United States-born status, nonmetropolitan residence, prepregnancy obesity, excess weight gain during pregnancy, and gestational diabetes. Examination of the factors that increase the risk for maternal hypertensive disorders along with the current interventions utilized to manage hypertensive disorders will assist in the identification of gaps in prevention and treatment strategies and implications for future practice. Specific focus will be placed on disparities among racially and ethnically diverse women that increase the risk for maternal hypertensive disorders. This review will serve to promote the development of interventions and strategies that better address and prevent hypertensive disorders throughout a pregnant woman's continuum of care.

## 1. Introduction

The World Health Organization defined maternal mortality as the death of a pregnant woman, regardless of the duration of the pregnancy, from any cause related to or exacerbated by the pregnancy, but not from accidental causes [1]. Since the Pregnancy Mortality Surveillance System was implemented, the number of reported maternal deaths in the United States as determined by the WHO's definition increased steadily from 7.2 deaths per 100,000 live births in 1987 to 18.0 deaths per 100,000 live births in 2014 [2]. This data presents a significant increase in maternal mortality rates in the United States and demands further investigation of underlying risks across diverse populations. While this documented rise in maternal mortality may be due in part to more rigorous and compelling reporting systems, it is critical that greater focus must be placed on developing innovative and individualized interventions and strategies to decrease the risk of maternal mortality among all populations.

Maternal mortality can be the result of a multitude of factors. Hypertensive disorders alone account for over a third of maternal deaths and contribute, in conjunction with other medical conditions, to half of all maternal deaths [3]. While not the focus of this minireview, studies have also identified an increase in chronic comorbid conditions during pregnancy as well, regardless of whether a hypertensive disorder is present or not [4]. Examples of comorbid conditions among pregnant women include diabetes and obesity, and when combined with a hypertensive disorder, these conditions place women at an even higher risk for adverse outcomes during pregnancy [4]. Furthermore, preexisting hypertension, renal disease, obesity, and collagen vascular disorders have also been identified as risk factors in the development of preeclampsia, a well-known hypertensive disorder in pregnancy [5]. While exercise and diet have long been recommended to address obesity and diabetes, it is important to note that not all environments are favorable for such lifestyle changes, especially when regarding access to healthy and fresh groceries and affordable places for fitness. Understandably, lower socioeconomic status is often associated with an increased prevalence of obesity [6]. Therefore, it is critical to consider the impact of social determinants of health on the risk for maternal hypertension. While acknowledging that chronic comorbid conditions are a crucial aspect of the contribution to maternal mortality, this minireview seeks to focus specifically on hypertensive disorders and their contribution to maternal mortality.

Hypertensive disorders alone occur in approximately 5 to 11% of pregnant women [6, 7]. Maternal hypertensive disorders include both chronic hypertension as well as gestational hypertension and preeclampsia [8–10]. The scope of this minireview will specifically focus on gestational hypertension. Ankumah and Sibai (2016), defined gestational hypertension as systolic blood pressure (BP) of 140 millimeters of mercury (mm Hg) and diastolic BP of 90 mm Hg documented either before pregnancy or before the 20th week of gestation on at least two separate occasions at least four hours apart [11]. Ideally, a woman with a diagnosis of hypertension under this definition should be evaluated before conception with emphasis on determining the cause of hypertension and achieving reasonable BP control before pregnancy occurs [7]. However, differentiating and managing a diagnosis of chronic hypertension from gestational hypertension may prove difficult as prepregnancy BP values are often not available. Lack of access to healthcare services and geographic location may impede regular clinical care prior to pregnancy, which may also result in a delay in the initiation of obstetric care. Women who develop gestational hypertension are at an increased risk of hypertension and stroke in later adult life which highlights the need for providers to monitor women during the postpartum period for resolution of BP elevation and conduct further workup for causes of hypertension if the retrospective diagnosis of gestational hypertension is not warranted [12].

Additionally, data illustrated that African American women contributed 14.6% of live births but 35.5% of maternal deaths and, therefore, are at a 3.2 times higher risk of dying from a pregnancy-related complication than non-Hispanic white women [1]. Furthermore, substantial racial and ethnic differences have also been noted in the prevalence of maternal hypertension: ranging from 2.2% for Chinese women and 2.9% for Vietnamese women to 8.9% for American Indian/Alaska Native women (AIANs) and 9.8% for African American women [6]. Thus, it is critical to better understand the intersection of maternal hypertensive disorders, racial and ethnic disparities, and their contribution to an increased risk of maternal mortality. Current practices related to the prevention and treatment of hypertensive disorders will be examined, creating an opportunity for the development of innovative approaches, both within and outside the clinical setting throughout the continuum of care.

## 2. Methods and Search Strategy

The exploration of the diagnosis and management of hypertensive disorders relating to an increased risk of maternal morbidity and mortality required an integrative literature review. Novel approaches that improve the prevention and early detection of hypertensive disorders were sought. An electronic search was conducted using databases and online search engines, including CINAHL, MEDLINE, EBSCO, and PubMed. The authors searched these databases for relevant studies using a combination of keywords, including hypertensive disorders, pregnancy, outpatient, clinical setting, racial disparities, and innovative approaches. The search criteria included English language studies from 2010 to 2019. Article abstracts were then screened for relevance using peer appraisal. When filtering articles, the authors specifically targeted literature that focused on race and ethnicity, hypertensive disorders, the impact of race on maternal hypertension, outcomes related to the experience of care, current practices and interventions for hypertensive disorders, and women's health. A final set of thirty studies were included for use in this minireview.

## 3. Discussion

The American College of Obstetricians and Gynecologists (ACOG) has published various guidelines for hypertensive disorders during pregnancy [13–15]. Specifically, the ACOG issued a report that included evidence-based recommendations for the prevention, diagnosis, and treatment of hypertension in pregnancy [13]. These hypertensive disorders can be categorized as (1) preeclampsia-eclampsia, (2) chronic hypertension, (3) chronic hypertension with superimposed preeclampsia, and (4) gestational hypertension [13]. The early prevention and detection of pregnancy-related hypertensive disorders are essential to monitor for new symptom development and to prevent complications [4]. Ankumah and Sibai outlined an example of the importance of early detection and prevention of complications which determined that the results of a preliminary workup for hypertensive disorders in the clinic can aid the clinician in classifying hypertensive women into low-risk and high-risk categories [11]. Although low-risk women tend to have excellent pregnancy outcomes, high-risk women are at an increased risk for poor maternal and neonatal outcomes. Consequently, high-risk women should be considered for an increased frequency of outpatient BP monitoring and clinic visits [7, 16].

Diagnosis of chronic hypertension at preconception or early in pregnancy is necessary as chronic hypertension is a recognized risk factor for preeclampsia, a hypertensive disorder with considerable risk for maternal mortality [9]. ACOG's recommendations illustrated that complications might arise when a pregnant woman with previously, undiagnosed hypertension initially presents to the clinic in the second trimester of pregnancy with normal BP, after having experienced the pregnancy-associated physiologic decrease in BP [9]. This woman will have been presumed to be normotensive upon assessment, and if her BP increased during the third trimester, she might be erroneously diagnosed with either gestational hypertension or preeclampsia [9]. This example highlights the need for intervention in the community setting, in the form of reproductive health education for women planning to become pregnant as well as an early clinical intervention for all women who are pregnant.

As evidenced by these examples, the current workup used to diagnose hypertensive disorders during pregnancy is typically conducted in the clinic setting and is based on a combination of symptom evaluation (including chest pain, headache, and epigastric pain), lab values, blood pressure values, and history [3]. Notably, the risk for maternal mortality related to hypertensive disorders increases for the populations without access to clinical care during pregnancy [6]. However, when a woman does have access to a clinical workup and is diagnosed with a hypertensive disorder during pregnancy, the essential goals of management are to prevent severe hypertension and cerebral events from developing. Often, the prevention of such developments is accomplished with the use of antihypertensive medications [5].

A study known as the CHIPS Randomized Controlled Trial (Control of Hypertension in Pregnancy Study) explored the effects of less-tight versus tight control of hypertension on pregnancy complications (less-tight control being a target diastolic blood pressure (dBP) of 100 mm Hg and tight control being a target dBP of 85 mm Hg). The study found no significant difference in the rates of complications or poor maternal outcomes between the two controls, but it was determined that the less-tight control was associated with a higher rate of severe maternal hypertension [17, 18]. Achieving tight control of maternal hypertension is difficult as studies have reported conflicting data related to the efficacy and timing of administering antihypertensive medication as a treatment for the management of maternal hypertensive disorders. For example, while low-dose aspirin prophylaxis should be considered for women with more than one moderate risk factors (maternal age, body mass index, and sociodemographic characteristics) for preeclampsia, antihypertensive therapies have not been illustrated to reduce the incidence of superimposed preeclampsia [5, 13, 19, 20].

Furthermore, as a result of the individual recommendations for the management of each type of hypertensive disorder, occasionally, the current treatment with antihypertensive medications is not indicated at all. Instead, for example, the ACOG and Folk both recommended the use of home blood pressure monitoring along with increased surveillance in the form of more frequent clinic visits to assess lab values and fetal growth for women with a diagnosis of low-risk gestational hypertension [3, 9]. In addition to at-home blood pressure cuffs, digital weight scales, phone oximeters, and mobile applications are additional technological solutions currently being utilized as interventions to monitor for hypertensive disorders among pregnant women. Despite current use, further evidence is required to support the use of these technologies as effective management methods for hypertensive disorders during pregnancy [4].

Despite the various interventions currently being used to treat hypertensive disorders, racial and ethnic disparities continue to contribute to maternal mortality [21–24]. The CDC defines disparities in health as differences in the burden of disease or in opportunities to achieve optimal health [25]. Gadson et al. explored the social determinants of racial and ethnic disparities as they relate to the utilization of prenatal care and subsequent maternal outcomes [26]. Results from the study found that African American, Hispanic, and Native American women were at risk for late entry into clinical care, and African American women alone were at a significantly higher risk for maternal mortality [26]. Delay in seeking prenatal care was further demonstrated in data from the Pregnancy Mortality Surveillance System, which presented that African American and Hispanic women who die of pregnancy-related causes are more likely than white women to initiate prenatal care in the second and third trimesters [1]. Factors that impact the utilization of clinical services during pregnancy, and subsequent health outcomes, include socioeconomic and cultural factors, accessibility of facilities, and discrepancies in quality care [2, 20].

Alhusen et al. examined factors that may influence delays in women seeking care [27]. These factors included experiences of institutional racism in both accessing and receiving prenatal care as well as elevated inflammatory markers of stress in women seeking care while in the presence of healthcare providers [21, 27]. Better knowledge of women's views towards accessing clinical care throughout the continuum of pregnancy can assist in the development of strategies to eradicate these barriers and potentially reimagine a care delivery model that serves pregnant women from all backgrounds. Importantly, further work is still necessary to meaningfully address these inequities.

## 4. Recommendations for Practice

**Figure 1:**
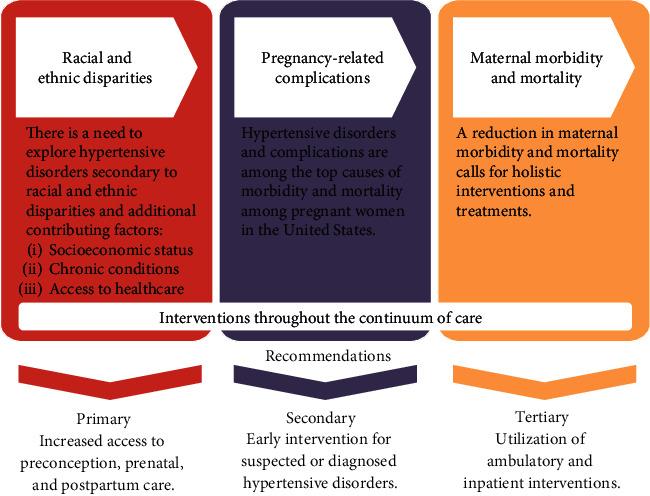
Primary, secondary, and tertiary level interventions and recommendations to address health disparities related to maternal hypertension.

Through the exploration of factors that increase the risk of maternal hypertension, it can be declared that addressing hypertensive disorders in pregnancy requires early identification. Ideally, the identification of hypertensive disorders would occur before pregnancy. Then, continuous, risk-appropriate clinical care and follow-up throughout the continuum of pregnancy could be implemented. This continuum of quality care is critical to improving maternal outcomes. To assist in the reduction of complications, there is a need for multifaceted interventions throughout both the intrapartum and postpartum periods [3]. Exploration and utilization of primary, secondary, and tertiary interventions should occur prior to conception to reduce maternal mortality related to hypertensive disorders [3, 28] (Figure 1). Furthermore, a priority should be placed on the development of interventions that are accessible to women located in both resource-rich and resource-poor settings during early pregnancy to address racial bias and discrimination in both the outpatient and clinic setting [14, 20]. Understanding the origins of these biases is an emerging public health priority considering the increasing rates of maternal mortality [16].

In addition to accessible interventions, successful management and prevention of maternal hypertensive disorders require consideration and inclusion of the social determinants of health during the development of interventions. It is necessary to acknowledge that risk factors for maternal hypertensive disorders extend beyond physical cause and are influenced by one's surrounding environment. Therefore, during intervention development and provision of education on maternal hypertensive disorders, it is paramount for providers to account for a woman's unique cultural values, beliefs, and socioeconomic status (SES) and environment of women of different races/ethnicities [29]. Therefore, additional research is recommended for the design and implementation of culturally appropriate and inclusive education to decrease and prevent the risk of maternal hypertensive disorders [29].

Along with clinical and outpatient recommendations, quality improvement (QI) recommendations need to be considered. Hernandez et al. identified contributing factors and missed opportunities relating to maternal mortality events in Florida and translated the findings into QI recommendations aimed at reducing maternal mortality [30]. The QI recommendations included (1) timely diagnosis and evidence-based treatment of specific clinical conditions including hypertensive disorders and (2) recognition and response to clinical triggers that show a change in clinical status [30].

## 5. Conclusions

Through this review, it can be determined that healthcare organizations should acknowledge that maternal mortality reflects maternal health among a population and may indicate gaps in care protocols in the clinical setting. In addition to implementing the clinical QI recommendations identified by Hernandez et al., it will be critical to develop and investigate at-home and community-based interventions that address earlier disparities in access to care [30]. Overwhelmingly, the literature in this minireview supports recommendations for further discussion and evaluation of the risks of hypertensive disorders that contribute to maternal mortality, while taking into consideration social determinants that may be influenced by the diversity, and the need for quality improvement interventions in and out of the clinic and throughout a woman's pregnancy.

## Abbreviations

ACOG: American College of Obstetricians and Gynecologists
AIANs: American Indian/Alaska Native women
BP: Blood pressure
mm Hg: Millimeters of mercury
QI: Quality improvement
WHO: The World Health Organization.
